# The immune-opioid axis in prediabetes: predicting prediabetes with insulin resistance by plasma interleukin-10 and endomorphin-2 to kappa-opioid receptors ratio

**DOI:** 10.1186/s13098-021-00677-w

**Published:** 2021-06-07

**Authors:** Shatha Rouf Moustafa

**Affiliations:** Clinical Analysis Department, College of Pharmacy, Hawler Medical University, Roya Towers C21, Erbil, Iraq

**Keywords:** Prediabetes, Insulin resistance, Interleukin, Endorphin, Endogenous opioid receptor

## Abstract

**Background:**

Prediabetes is characterized by a hemoglobin A1c of 5.7–6.4% and fasting blood glucose of 100–125 mg/dl. A high percentage of prediabetes subjects develop type 2 diabetes mellitus in the next years. The effects of opioid peptides and their receptors, in addition to immunological cytokines, on prediabetes are not well understood. Therefore, molecular, physiological, and clinical studies are required to link the opioid system, immune system, and insulin resistance (IR) in prediabetes. We hypothesize that opioid peptides (endomorphin-2 (EM2), and β-endorphin (βEP)), and their receptors (µ-opioid receptors (MOR) and κ-opioid receptors (KOR)), in addition to the inflammatory cytokines (IL-6) and anti-inflammatory cytokine (IL-10), affect IR parameters in patients with prediabetes.

**Methods:**

Sixty prediabetes patients with IR (prediabetes+IR) and sixty prediabetes patients without IR (prediabetes-IR), in addition to 58 controls, have participated in the study. IL-6, IL-10, EM2, βEP, MOR, and KOR were measured by the ELISA technique.

**Results:**

In general, most prediabetes subjects have dyslipidemia. The IL-6, IL-10, β-endorphin, MOR, and endomorphin-2 were higher in the prediabetes subgroups than the control group. The immune system was activated in the prediabetes in an IR-dependent manner. Prediabetes+IR can be predicted by the increased levels of IL-10, βEP, and EM2 and by the combination of IL-10 and EM2/KOR with good sensitivity and specificity.

**Conclusion:**

Opioid peptides and their receptors were upregulated in patients with prediabetes, depending on the significance of IR and the immune cytokines. The intercorrelation between the immune system, EOS, and insulin in prediabetes was confirmed.

## Background

Impaired fasting blood glucose (FBG) or glucose tolerance develops years before evolving into a strong type 2 diabetes mellitus (T2DM), and this disorder is known as prediabetes, a major risk factor for diabetes development [1]. About 34.5% of American adults over 18 years of age (88 million people) have prediabetes experience prediabetes, a disorder associated with higher hemoglobin A1c (HbA1c) and fasting blood glucose (FBG) levels than average but not significantly elevated to be grouped as diabetes mellitus [2]. Prediabetes is classified as HbA1c level between 5.7 and 6.4% and FBG between 100 and 125 mg/dl in the latest US guidelines [3].

Insulin resistance (IR) means the reduced sensitivity or reactivity of tissues to insulin-mediated biologic activity that leads to high glucose levels and represents the major risk factor of prediabetes and T2DM [4, 5]. IR, impaired insulin function, and hypersecretion of insulin are the main factors in prediabetes pathophysiology [6]. A large percentage of prediabetes patients showed a rise in IR index, and they labeled as insulin-resistant patients when the value of the homeostasis model assessment of the insulin resistance (HOMA2IR) reaches the cut-off value (> 2.5) [7, 8]. When blood glucose increases, it enters the hemoglobin and raises the HbA1c level that is considered as an indicator for β-cell function and the IR state [9]. There is evidence that connects insulin with the development of neurons and their normal functions. Insulin signaling is essential for the neurons' survival, learning, and memorization [10, 11]. Impaired insulin signaling in animal models leads to a collection or assembly of Aβ oligomers [12]. At formation, Aβ oligomers exhaust insulin receptors from the neuronal surface membrane, leading to IR and producing abnormal phosphorylation of the insulin receptor substrate (IRS) [13]. This state reduces the neurons' normal pro-survival signaling and promotes apoptosis to their death [14]. Notably, such structural network anomalies are related to the delay in processing information speed [15]. Elevated IR that occurs during midlife may increase the risk of cognitive impairment later in life, demonstrated by reduced verbal fluency and sluggish basic response time [16].

The relationship between IR parameters and endogenous opioid system (EOS) molecules and receptors that have many brain functions in some insulin-related disorders, including prediabetes, remains to be elucidated. EOS consists of peptides such as β-endorphin (βEP) and endomorphin-2 (EM2) and their receptors, µ-opioid receptors (MOR) and κ-opioid receptors (KOR). In addition to producing analgesia, opioids control glucose homeostasis by altering insulins' secretion [17]. There is evidence that stimulation of adrenal gland adrenoceptors can increase the secretion of β-endorphin, which stimulates peripheral MOR to alter glucose-associated genes, leading to improved peripheral glucose consumption and decreased hepatic gluconeogenesis for the improvement of extreme hyperglycemia [18]. The analysis of EOS peptides and receptors in animals and humans and their potential effect on insulin and glucagon release indicated that opioids might regulate insulin resistance and glucose metabolism [18, 19]. It is found that the MOR pathway is responsible for the improvement of insulin sensitivity [19]. Opioid receptors are found in the pancreas and the alpha and beta-pancreatic cells, thereby influencing endogenous opioid-mediated glucose and insulin homeostasis. Beta-endorphin has a strong relationship with the β-cell function and is considered an important predictor for differentiating between high and low β-cell functions [20]. Analysis of the isolated β-islets suggests that MOR directly mediates islet insulin hypersecretion and manages insulin release to the body compartments [21]. Opioids affect the function of β-cells in people who use heroin (MOR agonist), leading to increase blood glucose levels [22] and HbA1c [23]. The mechanisms behind these phenomena are increased use of glucose and decreased hepatic gluconeogenesis following improvement of peripheral MOR and heterogeneity of genes in glucose metabolism [18], in addition to the hyperglycemia caused by a chronic opioid receptor activation [24]. MOR mediates the inhibitory effects of EM2, which exercises a reduced role in diabetes. Besides, poor regulation of blood glucose can lead to the attenuated effects of EM2 [31].

The immune system molecules, particularly interleukin (IL)-6 and IL-10, are other important molecules that mediate the inflammatory response and need more study in prediabetes. Interleukin-6 (IL-6) is a pro-inflammatory cytokine that unequivocally motivates IR progression and T2DM pathogenesis via inflammation generation by regulating differentiation, migration, proliferation, and apoptosis of cells [25]. IL-6 has been examined since it is positively associated with the expression of the insulin-degrading enzyme (IDE), where the deficiency of IDE is related to obesity and T2DM [26]. Some results denote IL-6 's novel role in insulin metabolism, suggesting a mechanistic link between the IL-6 promotion and the IR promotion [25]. IL-10 is a cytokine with anti-inflammatory properties, modulating inflammatory responses by repressing the generation of pro-inflammatory cytokines [27]. Serum IL-10 has a converse relationship with hyperinsulinemia and IR, as IL-10 decreases with HOMA-IR increased [28].

This study hypothesized that opioid peptides and their receptors combined with the immune cytokines (IL-6 and IL-10) affected the IR parameters in prediabetes patients. The null hypothesis is a lack of intercorrelation between all the three systems and no correlation between prediabetes and these systems. This work's findings may potentiate the pharmacological intervention by targeting IR through the EOS components and immune biomarkers. To examine these hypotheses, we measured serum levels of some opioids proteins and receptors in addition to IL-6 and IL-10 levels in prediabetes patients who have/have not an IR state and compared with the healthy controls.

## Subjects and methods

### Subjects

More than 500 subjects, who check FPG routinely in the laboratories, were examined to select our study group with restricted criteria. A total of 120 subjects with prediabetes were chosen to participate in the study. A physician diagnosed these subjects under the American Diabetes Association’s criteria (FPG = 5.55–6.94 mM, HbA1c = 5.7–6.4%). The samples were collected from the Rizgari Teaching Hospital and private clinics and laboratories in Erbil City, Kurdistan Region, Iraq, from October 2019 to December 2019. All procedures were conducted following the established ethical standards. All study subjects provided written informed consent before participation in the study. The study was carried out under the international and Iraq ethics and privacy laws and approved by the Ethics Committee of Medical Research at the College of Pharmacy/Hawler Medical University. The reference No. of the ethical approval paper is HMU-PH-EC 191223/102. All procedures were performed according to the Helsinki Declaration's ethical standards for experiments involving humans, as revised in 2013.

The subjects with prediabetes were further divided into two subgroups following the results of HOMA2IR. The first group, prediabetes+IR, comprised subjects with a high IR state (HOMA2IR > 2.5). The second group, prediabetes−IR, comprised subjects with a low IR state (HOMA2IR < 2.5). This classification occurred deliberately in the same number of subjects in the two groups to remove the number of cases' possible bias. Fifty-eight healthy subjects were selected as the control group. Age ranges and sex ratios were matched in all the study groups. None of these subjects manifested any evident systemic disease or took drugs. Furthermore, in all subjects, the C-reactive protein (CRP) was negative (lower than 6 mg/l) to exclude overt inflammation. Tobacco use disorder (TUD) was examined under the DSM-IV-TR criteria. Body mass index (BMI) was calculated using the formula: body weight (kg)/squared height (m^2^). Subjects performing more than 30 min of moderate activity 2–3 times/week and never or less than one time per week were considered as a person with physical activity [3].

### Exclusion criteria

The present study excluded patients who met the following criteria: serum TG > 5.32 mM patients to satisfy the Friedewald’s formula, FPG > 25 mM, and fasting insulin > 400 pM to satisfy the HOMA calculator software requirements. Any patient with apparent diabetes mellitus, heart disease, hypertension, and those taking lipid-lowering drugs (e.g., simvastatin or atorvastatin) and metformin was excluded. We also excluded any subject with a urinary albumin/creatinine of more than 30 mg/g to exclude microalbuminuria, which indicated damage to the microvessels.

### Measurements

After at least 12 h of fasting, blood samples were collected in the morning and transferred into a plain tube and EDTA tubes. After clotting, sera samples were separated and divided into three aliquots and stored in a refrigerator before use. The serum glucose, total cholesterol, and TG were measured using commercially available kits supplied by Spinreact®, Spain. The absorbances were measured by using a visible spectrophotometer (model 722,

Shanghai Lianhua Industrial Co. Ltd., China). Serum HDLc was measured after precipitation of other lipoproteins using a reagent containing sodium phosphotungstate and magnesium chloride. The cholesterol content of the supernatant was measured using a cholesterol kit. VLDLc was determined using Friedewald’s equation (LDLc = Tc − HDLc − VLDLc), based on TG/2.19 and LDLc. The percentage of HbA1c in the whole blood (EDTA tube aliquot) and the urinary albumin/creatinine ratio were measured using the immunofluorescence analyzer (Finecare™ II FIA Meter, Guangzhou Wondfo Biotech Co., Ltd, China). The normal range of the HbA1c kit was 4–6%, and the microalbumin ratio was less than 30 mg/g. The IR parameters were calculated from the fasting glucose and insulin concentrations using the HOMA calculator program (http://www.dtu.ox.ac.uk/HOMA-calculator/download.php). The insulin resistance function (HOMA2IR), insulin sensitivity (HOMA2%S), and β-cell function (HOMA2%B) indices were generated using this software. An ideal person of average weight aged < 35 years had 1 HOMA2IR and 100% HOMA type β-cell activity. The serum insulin level was assayed using a solid-phase enzyme-linked immunosorbent assay (ELISA) kit based on the sandwich principle supplied by Calbiotech®, China. Other biomarkers and their suppliers were IL-10 (Elabscience®, Inc. CA, USA), MOR, KOR, and EM2 (Mybiosource®, Inc. CA, USA), and IL-6 and βEP (Melsin Medical Co, Jilin, China). The sensitivities of β-endorphins, MOR, KOR, endomorphin-2, and IL-6 ELISA kits were 0.1 pg/ml, 7.18 pg/ml, 1.0 ng/ml, 0.33 pg/ml, and 0.1 pg/ml, respectively. All measured concentrations were greater than their assay sensitivities. All intra-assay coefficients of variation were < 10.0%. The absorbances of the microplates were measured by a microplate reader supplied by BioTek, Guangzhou, China. Serum CRP was measured using a kit supplied by Spinreact®, Spain, using a test based on the latex agglutination principle.

### Statistical analysis

Analysis of variance (ANOVA) was employed to assess the differences in all measured biomarkers between diagnostic categories, and the χ^2^ test was used to compare the proportions and nominal variables. The associations among variables were computed using Pearson’s product-moment and Spearman’s rank-order correlation coefficient. The multivariate general linear model (GLM) analysis was used to delineate the effects of diagnosis for the prediabetes+IR, prediabetes−IR, and control groups while controlling the background variables, including age and sex. Protected LSD tests were used to check pairwise comparisons among treatment means. The model-generated estimated marginal mean (SE) values were computed after adjusting for covariates. Multiple regression analysis was used to delineate the significant biomarkers associated with the prediabetes+IR, and the results were checked for multicollinearity (tolerance and VIF values) and homoscedasticity (White and Breusch–Pagan tests). The binary logistic regression analysis was used to delineate the essential explanatory variables that predict prediabetes (versus control as the reference group). The data were subjected to ln transformation to normalize the measured biomarkers' data distribution (tested using the Kolmogorov–Smirnov test). However, the nonlinearity of any biomarker's mean and variance is a predictable source of variability that is eliminated using *z* scores. The natural logarithm of the relevant *z* unit scores was computed to transform the nonparametric variables into normally distributed components and apply the statistical analysis as a linear group. All tests were two-tailed, and a p-value < 0.05 was used for statistical significance. All statistical analyses were performed using the IBM SPSS windows version 25, 2017. Odds ratios (OR) and 95% confidence intervals (CI) for unfavorable glycemic status by study factors were calculated.

## Results

### Sociodemographic data

Table 1 shows the sociodemographic data of the prediabetes−IR, prediabetes+IR, and healthy control groups. No significant difference was observed in age, BMI, sex ratio, and marital status among the study groups.

**Table 1:** Demographic and clinical data of healthy controls (HC) and Prediabetes-IR versus Prediabetes+IR groups

Parameter	Control^A^ N = 58	Prediabetes-IR^B^ N = 60	Prediabetes + IR^C^ N = 60	F/χ^2^	df	p
Sex (F/M)	28/30	28/32	27/33	0.364	2	0.552
Age year	36.67 ± 10.03	35.03 ± 11.44	37.9 ± 11.88	0.499	2/175	0.609
BMI kg/m^2^	26.39 ± 3.72	27.76 ± 4.01	27.63 ± 5.14	0.909	2/175	0.407
Smoking (Y/N)	17/41	18/42	19/41	0.321	2	0.714
Marital status (M/S)	33/25	31/29	36/24	1.308	2	0.067
Physical activity (Y/N)	21/37	20/40	18/42	1.412	2	0.056
TC mM	4.07 ± 0.59^C^	4.42 ± 0.56	4.72 ± 0.70^A^	8.288	2/175	0.001
TG mM	1.40 ± 0.14^C^	1.49 ± 0.17	1.57 ± 0.36^A^	3.701	2/175	0.029
VLDLc mM	0.64 ± 0.07 ^C^	0.68 ± 0.08	0.72 ± 0.16^A^	3.701	2/175	0.029
HDLc mM	1.12 ± 0.11^C^	1.06 ± 0.14	0.99 ± 0.13^A^	6.789	2/175	0.002
LDLc mM	2.32 ± 0.60^C^	2.69 ± 0.58	3.01 ± 0.74^A^	8.725	2/175	< 0.001
TC/HDLc	3.69 ± 0.64^B,C^	4.27 ± 0.81^A,C^	4.81 ± 0.80^A,B^	16.512	2/175	< 0.001
TG/HDLc	1.27 ± 0.19^C^	1.44 ± 0.26	1.61 ± 0.42^A^	9.298	2/175	< 0.001
LDLc/HDLc	2.11 ± 0.60^B,C^	2.61 ± 0.75^A,C^	3.07 ± 0.81^A,B^	13.214	2/175	< 0.001
zLn IL-6	− 0.35 ± 1.15^B,C^	0.16 ± 0.90^A^	0.19 ± 0.86^A^	4.562	2/175	0.019
IL-10 pg/ml	7.9 ± 2.27^B,C^	11.46 ± 4.52^A^^,C^	14.08 ± 8.19^A,B^	9.362	2/175	< 0.001
zLn βEP	− 0.50 ± 0.39^B,C^	0.37 ± 1.39 ^A^	0.13 ± 076^A^	6.834	2/175	0.002
zLn MOR	− 0.34 ± 1.15^B,C^	0.19 ± 0.96^A^	0.15 ± 0.81^A^	4.087	2/175	0.024
zLn (βEP/MOR)	− 0.09 ± 1.11	0.10 ± 1.02	0.01 ± 0.87	0.279	2/175	0.757
zLn EM2	− 0.50 ± 0.89^B,C^	0.16 ± 0.98^A^	0.34 ± 0.964^A^	6.615	2/175	0.002
zLn KOR	− 0.35 ± 1.20^B,C^	− 0.21 ± 1.14^A^	0.14 ± 0.64^A^	3.964	2/175	0.031
zLn (EM2/KOR)	− 0.15 ± 1.14	− 0.038 ± 0.94	0.19 ± 0.90	0.888	2/175	0.415
HbA1c %	5.01 ± 0.43^B,C^	6.02 ± 0.2^A^	6.17 ± 0.2^A^	132.746	2/175	< 0.001
Glucose mM	5 ± 0.71^B,C^	5.92 ± 0.59^A,C^	6.49 ± 0.39^A,B^	49.734	2/175	< 0.001
Insulin pM	68.54 ± 10.79^B,C^	100.74 ± 13.64^A,C^	140.48 ± 12.38^A,B^	256.628	2/175	< 0.001
I/G mM	14.07 ± 3.40^B,C^	17.12 ± 2.46^A,C^	21.79 ± 2.84^A,B^	52.921	2/175	< 0.001
zLnHOMA2%B	0.13 ± 1.39	0.18 ± 0.84	0.05 ± 0.63	0.764	2/175	0.469
HOMA2%S	80.72 ± 13.82^B,C^	52.63 ± 7.51^A,C^	36.85 ± 2.80^A,B^	174.254	2/175	< 0.001
HOMA2IR	1.27 ± 0.19^B,C^	1.94 ± 0.27^A,C^	2.73 ± 0.22^A,B^	303.865	2/175	< 0.001

^A,B,C^ Pairwise comparisons between group means

zLn: z-score of the natural logarithm; FPG: fasting plasma glucose; TC: serum total cholesterol; TG: serum triglycerides; VLDLc: very-low-density lipoproteins; HDLc: high-density lipoproteins; LDLc: Low-density lipoproteins; IL: interleukin; KOR: κ-opioid receptor; MOR: µopioid receptor; EM2: Endomorphin-2; βEP: β-endorphin; BMI: Body mass index; HOMA2IR: homeostasis model assessment 2 of insulin resistance; HOMA2%S: homeostasis model assessment 2 of insulin sensitivity percentage; HOMA2%B: homeostasis model assessment 2 of beta-cell function percentage; IR: insulin resistance (HOMA2IR > 2.5)

A significant increase (p < 0.05) in TC, TG, VLDLc, and LDLc and a significant decrease in HDLc was observed in the prediabetes+IR group compared with the control group. No such difference was observed between the prediabetes−IR and the prediabetes+IR groups. The atherogenic indices (TC/HDLc and LDLc/HDLc) were significantly different among the three study groups, and the scores followed the order: prediabetes+IR > prediabetes−IR > controls. The TG/HDLc showed a significant increase in the prediabetes+IR group compared with the control group.

No significant difference was observed in the levels of the ratios of opioids to their receptors zLn (βEP/MOR) and zLn (EM2/KOR) among the study groups. The level of other opioids in the prediabetes groups was higher than that in the control group: zLn βEP (F = 6.834, df = 2/175, p = 0.002), zLnMOR (F = 4.087, df = 2/175, p = 0.024), zLnEM2 (F = 6.615, df = 2/175, p = 0.002), and zLnKOR (F = 3.964, df = 2/175, p = 0.031).

Serum IL-10 was significantly different among the three study groups (F = 9.362, df = 2/175, p < 0.001), and the score followed the order: prediabetes+IR > prediabetes−IR > controls. zLnIL-6 was significantly increased in the prediabetes groups than the control group, whereas no such difference was observed between the prediabetes subgroups.

The IR parameters, namely, glucose (F = 49.734, df = 2/175, p < 0.001), insulin (F = 256.628, df = 2/175, p < 0.001), I/G (F = 52.921, df = 2/175, p < 0.001), HOMA2%S (F = 174.254, df = 2/175, p < 0.001), and HOMA2IR (F = 303.865, df = 2/175, p < 0.001), followed the order: controls < prediabetes−IR < prediabetes+IR. The HbA1c% in the prediabetes−IR and the prediabetes+IR groups was higher than that in the control group (F = 132.746, df = 2/175, p < 0.001). zLnHOMA2%B showed no significant difference among the study groups (F = 0.764, df = 2/175, p = 0.469).

### Differences in the biomarkers between the study groups

In the entire study group, significant correlations were observed between the zLnMOR and the following parameters: IL-10 (r = 0.306, p = 0.017), zLnKOR (r = 0.311, p = 0.016), and zLnIL-6 (r = 0.368, p = 0.004). zLnKOR was correlated with IL-6 (r = 0.451, p < 0.001) and zLnEM2 with (r = 0.377, p = 0.003).

### Multivariate GLM analysis

Table 2 displays the multivariate GLM analysis outcomes comparing the measured biomarkers' differences among the three study groups while adjusting for age, BMI, sex, physical activity, IR parameters, and smoking. Significant differences (p = 0.040) were observed in the biomarkers among the groups with an effect size of 0.198, whereas the other covariates had no significant effects (p > 0.05). The tests for between-subject effects in Table 2 and the results in Table 3 showed the SE values and indicated that all eight biomarkers of the patients with prediabetes were significantly higher than those of the control group. Furthermore, the IL-10, zLnβEP, zLnMOR, and zLnEM2 in the prediabetes subgroups were significantly higher than those in the control group. Among all the examined biomarkers, zLnβEP had the highest effect on the diagnosis of prediabetes (F = 5.128, df = 2/175, p = 0.027, partial η^2^ = 0.067).

**Table 2:** Results of multivariate GLM analysis showing the associations between biomarkers and diagnosis of prediabetes while adjusting for background variables

Type	Dependent variable	Explanatory variable	F	df	p	Partial η^2^
Multivariate	IL-10, zLn βEP, zLn (βEP/MOR), zLn IL-6, zLnMOR, zLnEM2, zLnKOR, zLn (EM2/KOR)	Diagnosis	2.203	16/212	0.045	0.192
		Sex	0.450	8/156	0.867	0.046
		Age	1.138	8/156	0.351	0.109
		BMI	1.803	8/156	0.102	0.163
		Smoking	0.604	8/156	0.750	0.061
		Physical activity	0.798	8/156	0.612	0.084
		HbA1c	1.076	8/156	0.389	0.104
		Glucose	0.801	8/156	0.590	0.079
		Insulin	0.851	8/156	0.550	0.084
		I/G	0.968	8/156	0.462	0.094
		HOMA2%S	0.495	8/156	0.835	0.051
		HOMA2IR	0.841	8/156	0.558	0.083
		zLnHOMA2%B	1.177	8/156	0.328	0.112
		Dyslipidemia	0.020	8/156	0.966	0.001
Tests of Between-Subjects Effects	Diagnosis	IL-10	2.102	2/163	0.152	0.029
	Diagnosis	zLnβEP	5.128	2/163	0.027	0.067
	Diagnosis	zLn(βEP/MOR)	0.884	2/163	0.350	0.012
	Diagnosis	zLn IL-6	0.202	2/163	0.654	0.003
	Diagnosis	zLnMOR	1.131	2/163	0.291	0.016
	Diagnosis	zLnEM2	0.003	2/163	0.957	0.001
	Diagnosis	zLnKOR	3.745	2/163	0.057	0.050
	Diagnosis	zLn (EM2/KOR)	2.213	2/163	0.141	0.030

zLn: z-score of the natural logarithm; FPG: fasting plasma glucose; IL: interleukin; KOR: κ-opioid receptor; MOR: µopioid receptor; EM2: Endomorphin-2; βEP: β-endorphin

**Table 3:** Model-generated estimated marginal means values (SE) of the biomarkers in prediabetes (versus healthy controls) and prediabetes−IR versus prediabetes+IR and healthy controls

Biomarkers	Control^A^	Prediabetes-IR^B^	Prediabetes+IR^C^
IL-10	7.981 (1.062)^B,C^	11.461 (1.026)^A^	14.082 (1.026)^A^
zLn βEP	12.146 (2.308)^B,C^	23.652 (2.230)^A^	20.638 (2.230)^A^
zLn (βEP/MOR)	5.362 (0.677)	5.976 (0.654)	5.572 (0.654)
zLn IL-6	0.314 (− 0.187)	0.157 (0.180)	0.194 (0.180)
zLnMOR	0.431 (− 0.180)^B,C^	0.194 (0.174)^A^	0.149 (0.174)^A^
zLnEM2	0.518 (− 0.180)^B,C^	0.163 (0.174)^A^	0.338 (0.174)^A^
zLnKOR	0.362 (− 0.186)	0.213 (0.180)	0.140 (0.180)
zLn (EM2/KOR)	0.158 (− 0.191)	0.038 (− 0.185)	0.188 (0.185)

^A,B,C^ Pairwise comparisons between group means

zLn: z-score of the natural logarithm, IL: interleukin; KOR: κ-opioid receptor; MOR: µopioid receptor; EM2: Endomorphin-2; βEP: β-endorphin

Table 4 shows the results of two binary logistic regression analyses examining the best predictors of prediabetes (versus controls) and prediabetes−IR (versus prediabetes+IR) by using an automatic stepwise method with biomarkers as explanatory variables while allowing the effects of other cofounders (age, sex, and smoking). The first regression analysis showed that prediabetes was best predicted by increased levels of IL-10, zLnβEP, and zLnEM2 (χ^2^ = 38.122, df = 7, p < 0.001, Nagelkerke = 0.480) with an accuracy of 76.7%, sensitivity of 85.0%, and specificity of 75.6%. The second regression analysis showed that the combination of IL-10 and zLnEM2/KOR were the best predictors of prediabetes+IR versus prediabetes−IR (χ^2^ = 14.780, df = 7, p = 0.031, Nagelkerke = 0.364) with an accuracy of 71.2%, sensitivity of 74.4%, and specificity of 72.7%.

**Table 4:** Results of two different binary logistic regression analyses with prediabetes (versus healthy controls) and Prediabetes-IR versus Prediabetes+IR as dependent variables and the biomarkers as explanatory variables

Dichotomies	Explanatory variables	B	SE	Wald	df	p	OR	95% CI
Prediabetes/Controls	IL-10	0.359	0.128	7.876	1	0.005	1.431	1.114–1.839
	zLnβEP	0.316	0.137	5.311	1	0.021	1.372	1.048–1.795
	zLnEM2	0.846	0.396	4.569	1	0.033	2.331	1.073–5.063
Prediabetes-IR/Prediabetes+IR	IL-10	0.618	0.05	2.505	1	0.043	1.083	0.981–1.195
	zLnEM2/KOR	0.555	0.433	4.846	1	0.024	2.611	1.187–3.947

zLn: z-score of the natural logarithm; IL: interleukin; KOR: κ-opioid receptor; MOR: µ-opioid receptor; EM2: Endomorphin-2; βEP: β-endorphin; IR: insulin resistance (HOMA2IR > 2.5)

### Prediction of symptom domains by biomarkers

Table 5 shows different stepwise multiple regression analyses with the IR parameters as dependent variables and the eight biomarkers as explanatory variables while allowing the effects of age and sex. Regression #1 showed that the regression could explain 21.1% of the variance in the total FPG on IL-10, zLnKOR, and zLn (EM2/KOR). Regressions #2, #4, #5, and #6 showed that the same variables explained a considerable part of the variance in insulin (22.4%), HbA1c (29.0%), HOMA2IR (29.3%), and HOMA2%S (29.7%). Regression #3 showed that 13.0% of the variance in the I/G ratio was explained by IL-10.

**Table 5:** Results of multiple regression analysis with IR parameters as dependent variables and biomarkers as explanatory variables

Dependent variables	Explanatory variables	β	t	p	F _model_	df	p	R^2^
1. FPG	Model				3.142	7/82	0.005	0.211
	IL-10	0.279	2.661	0.009				
	zLnKOR	0.374	2.625	0.010				
	zLn(EM2/KOR)	0.302	2.537	0.130				
2. Insulin	Model				4.669	7/82	< 0.001	0.224
	IL-10	0.397	3.973	< 0.001				
	zLnKOR	0.339	2.498	0.015				
	zLn(EM2/KOR)	0.271	2.393	0.019				
3. I/G	Model				2.907	7/82	0.009	0.130
	IL-10	0.322	3.041	0.003				
4. HbA1c	Model				6.200	7/82	< 0.001	0.290
	IL-10	0.318	3.333	0.001				
	zLnKOR	0.386	2.977	0.004				
	zLn(EM2/KOR)	0.356	3.284	0.002				
5. HOMA2IR	Model				4.809	7/82	< 0.001	0.293
	IL-10	0.401	4.029	< 0.001				
	zLnKOR	0.355	2.624	0.010				
	zLn(EM2/KOR)	0.280	2.485	0.015				
6. HOMA2%S	Model				4.836	7/82	< 0.001	0.297
	IL-10	− 0.348	− 3.497	0.001				
	zLnKOR	− 0.371	− 2.749	0.007				
	zLn(EM2/KOR)	− 0.290	− 2.577	0.012				

zLn: z-score of the natural logarithm, FPG: fasting plasma glucose, IL: interleukin; KOR: κ-opioid receptor; MOR: µopioid receptor; EM2: Endomorphin-2; βEP: β-endorphin; HOMA2IR: homeostasis model assessment 2 of insulin resistance, HOMA2%S: homeostasis model assessment 2 of insulin sensitivity percentage; HOMA2%B: homeostasis model assessment 2 of beta-cell function percentage

The subjects with prediabetes had dyslipidemia, and not all of them underwent the IR state. The IL-6, IL-10, βEP, MOR, and EM2 were higher in the prediabetes. MOR was correlated with IL-10 and KOR. Prediabetes+IR can be predicted by the increased levels of IL-10, βEP, and EM2 and by the combination of IL-10 and EM2/KOR with good sensitivity and specificity.

## Discussion

The first significant result is the increased atherogenic indices and dyslipidemia state in the prediabetes +IR group compared to the controls, indicating the negative effect of elevated IR on lipid metabolism (Table 1). IR may also alter systemic lipid metabolism and endothelial dysfunction that ultimately contribute to dyslipidemia and atherosclerotic plaque formation [29, 30]. IR in the myocardium causes damage through a modification of the signal transduction, impaired regulation of the substrates' metabolism, and variation in the delivery of myocardium substrates [29, 31]. These heart muscles' changes are associated with the presence of increased IR in the prediabetes patients and absent in the prediabetes-IR group.

**Fig. 1:**
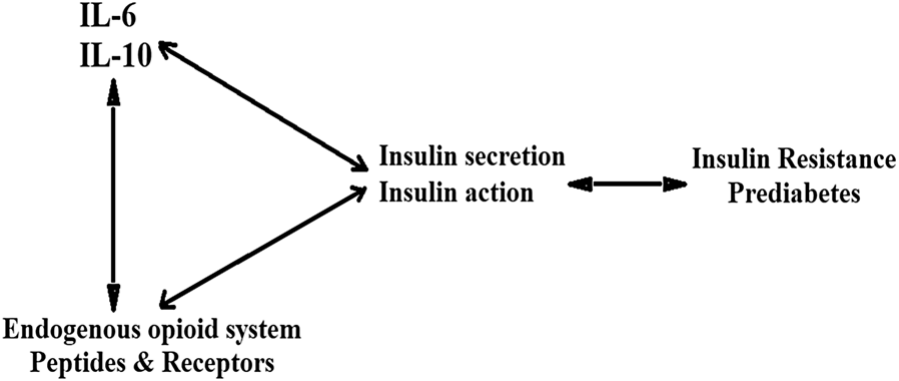
Potential mutual interaction between immune system, EOS peptides and insulin secretion and synthesis

The increase of the endogenous opioids and their receptors in the prediabetes subgroups compared with the controls suggests the dependence of these parameters on the state of the glucose metabolism rather than its dependence on the insulin hormone response. To our knowledge, these results are the first study that measured endogenous opioids in prediabetes disorder. Previous research examined the IR parameters with opioid peptides and receptors in other diseases [19, 32, 33]. However, there is no definite explanation for the associations between IR and EOS molecules. Among the suggested explanations, MOR is enhanced in insulin-sensitive tissues, such as the skeletal muscle, resulting in a reversal of insulin-stimulated glucose disposal impairment in genetically obese rats through exercise training [19]. This IR enhancement is associated with increased ß-endorphin secretion, thereby enhancing the post-receptor and insulin signaling cascade, including the downstream phosphatidylinositol-3 kinase signaling pathway involved in glucose translocation [18, 19]. Interestingly, in the IR stage, brain damage and disturbance of white matter occur without overt diabetes, and these structural changes may cause early cognitive dysfunction [34] and secrete endogenous peptides into the bloodstream and increase their rates. The hypothesis is the mutual interaction between pro-/ inflammatory cytokines, EOS peptides, and their receptors with insulin secretion and synthesis, as seen in Fig. 1.

EOPs administer the β-cell endocrine function resulted in insulin secretion through paracrine and intracrine mechanisms within the pancreas [21, 35]. Increasing the release of βEP from the adrenal gland may stimulate peripheral opioid MOR to increase muscle glucose transporter expression and/or reduce gene-level hepatic gluconeogenesis, thereby increasing the use of glucose in peripheral tissues to improve severe hyperglycemia [18]. Therefore, increased opioid production locally results in the release of insulin from the pancreas. IR results from hepatic insulin receptor downregulation, thereby increasing the insulin levels in peripheral blood [5, 17].

The peptides and receptors of EOS in the brain areas control appetite [36] and mood and are associated with mood disorders [37]. MOR and KOR appear to compensate for the inclusion by the nucleus of specific food intakes [38]. Activation of β-cells and subsequent regulation of insulin secretion and glucose metabolism by MOR is mediated via sympathetic innervation [39]. Treatment with the selective KOR agonist diminishes the blood glucose level dramatically [40].

For prediabetes subjects, the elevation of EM2 could be due to an increase in their release in response to beta cells' potential injury. EM2 can preserve the beta-cell islets in animal studies from the injury caused by streptozotocin, alloxan, and hydrogen peroxide [40]. EM2 improves islet viability and increases the cell supernatant's insulin production after streptozotocin and stimulation by alloxan. These results indicated that endomorphins might have protective effects on oxidative injury to islet cells [40].

The prevalence of these differences in prediabetes groups and controls should be considered when finding a new treatment target to prevent prediabetes from progressing into T2DM. Between the three study groups, serum IL-10 was significantly different, and the score followed the order: controls < prediabetes−IR < prediabetes+IR. In a previous study, relative to healthy controls [41], the serum IL-10 level is elevated in T2DM but not in prediabetes.

The present study has excluded the patients with positive CRP to ensure we have included only the patients free from overt inflammation. However, several studies documented the introduction of the Th1 cell subset and the causal involvement of this phenomenon in inflammation and IR in the diabetes mouse models [42]. In prediabetes groups, IL-6 was significantly elevated compared to the control group, whereas there was no such difference between the prediabetes subgroups. IL-6 showed no significant difference between the groups and controls for prediabetes [43]. Nonetheless, hyperglycemic/hyperinsulinemic conditions [43] show increased IL-6, another pro-inflammatory marker. The findings showed significant associations between IL-10 and MOR, as well as between IL-6 and KOR. Exogenous opioids inhibit the ability of macrophages, natural killer cells, and T-cells to in-vitro and animal models weaken the gut barrier [44]. Immune cells secrete endogenous opioid peptides connected to peripheral opioid receptors to alleviate inflammatory pain. The immune system and endogenous opioids are usually similarly co-operative [44]. IL-10 and MOR have a depression relationship [37], suggesting that, in addition to the possible correlation between immune and opioid systems, mood triggers such an interaction.

The second significant result was obtained from the multivariate GLM study, which excluded the covariate effects (age, BMI, sex, IR parameters, physical activity, and smoking) to compare the parameters according to the diagnostic form (Table 2). Around 20% (0.198) of immune—opioid biomarker values can be clarified by prediabetes and IR. Based on Tables 2 and 3 (SE) findings, all eight biomarkers in patients with prediabetes were significantly higher compared with the controls. Of all of the biomarkers tested, zLnβEP had the most significant effect on prediabetes diagnosis. βEP cells have been seen in areas near pancreatic β-cells, and opioids increase insulin secretion [45]. Increased circulating βEP is associated with enhancing IR to ameliorate the post-receptor insulin signaling cascade and enhance insulin sensitivity through peripheral motivation [19]. By sharing insulin release, MOR controls body weight, showing a novel target for new diabetes treatments [21]. The endogenous opioid system in vitro negatively regulates insulin secretion from isolated islets of Langerhans [46]. Additionally, some groups recorded the dual stimulative/inhibitory effect of β-endorphin on insulin secretion depending on dose, obesity, or circulating glucose level [47].

MORs function as a part of the complex opioid mechanism, mediating the effects of endogenous opioids such as EM2 and various exogenous opioid agonists [48]. MOR participates in glucose homeostasis by adversely controlling glucose tolerance by inhibiting insulin release from the β-cell and the β-cell mass [21]. In muscle tissues and cells, an association occurs between MOR and IRS [32]. It is found that opioid-induced insulin secretion may be based on G-protein independent mechanisms [49]. Based on a linear regression analysis, βEP is the strongest protein associated with the β-cell function [20].

The analyzes of binary logistic regression in Table 4 showed that the increased rates of the combination of IL-10, zLnβEP, and zLnEM2 could be used with reasonable precision to distinguish prediabetes+IR from other classes (76.7%). Another collection of increased IL-10 and zLnEM2 / KOR can be used with reasonable precision (71.2%) to predict prediabetes+IR. These findings further indicate the value in prediabetes+IR of these parameters and the close association with the immune-opioid system. EOS molecule production increases by acting on the pancreatic β-cells, increasing insulin secretion resulting in elevated insulin levels in the hepatic circulation. Increased insulin levels contribute to hepatic insulin receptors' downregulation, triggering, and sustaining hyperinsulinemia [7, 19]. Considering that MOR's central stimulation impairs glucose tolerance and responsiveness to insulin and motivates hepatic gluconeogenesis [50], stimulation of peripheral MOR can improve IR in animals and provide a novel target for IR treatment [51]. MOR activation increases IL-6-induced IR by contrasting insulin sensitivity by specific insulin signals [52]. In contrast, KOR activation results in decreased levels of IL-6 [53].

The effect of the calculated biomarkers on the IR parameters is shown in Table 5. The regression on IL-10, zLnKOR, and zLn(EM2/KOR) will explain approximately one-fifth (21.1%) of the variance in FPG. In subjects with obesity, elevated endogenous opiates can influence the insulin response to glucose through impaired or standard oral glucose tolerance tests [54]. The same changes explain a significant portion of the insulin variations, HbA1c, HOMA2IR, and HOMA2%S. It is found that the decrease in the IR condition following a decrease in body weight has no significant effect on the βEP level [55]. The independent parameters (FBG, leptin, and HbA1c) linked well in healthy people because the beta cells were functioning correctly. Elevated blood glucose induces insulin concentrations’ elevation when released from the healthy beta cells to maintain average blood glucose concentrations over long periods [56].

## Conclusion

Most prediabetes subjects had an IR state, an elevation in the immune and opioid biomarkers, and dyslipidemia. Of all the biomarkers tested, βEP has the highest diagnostic value for prediabetes. The immune system was activated in the prediabetes in an IR-dependent manner. Prediabetes+IR can be predicted using the increased rates of the IL-10, βEP, and EM2 mixture and a mixture of IL-10 and EM2/KOR with strong sensitivity and specificity. Therefore, we rejected the null hypothesis and confirmed intercorrelation between the immune system, EOS, and insulin in prediabetes.

## Limitations of the study

The major limitation of the study is the relatively low number of participants. As no institution funded the work, the author could not measure other cytokines, opioids, and receptors to obtain a whole picture of these parameters' role in prediabetes.

## Abbreviations

ANOVA: Analysis of variance
βEP: β-Endorphin
BMI: Body mass index
CI: Confidence intervals
CRP: C-reactive protein
DSM-IV-TR: Diagnostic and statistical manual of mental disorders, fourth edition, text revision
ELISA: Enzyme-linked immunosorbent assay
EM2: Endomorphin-2
FBG: Fasting blood glucose
EOS: Endogenous opioid system
GLM: General linear model
GLUT4: Glucose transporter 4
HbA1c: Glycated hemoglobin
HDLc: High-density lipoprotein cholesterol
HOMA2: Homeostasis model assessment version 2
HOMA%B: Beta-cell function percentage
HOMA%S: Insulin sensitivity percentage
IDE: Insulin-degrading enzyme
IL: Interleukin
IR: Insulin resistance
IRS: Insulin receptor substrate
KOR: κ-Opioid receptor
LDLc: Low-density lipoprotein cholesterol
MOR: µ-Opioid receptor
OR: Odds ratios
SE: Standard error
Tc: Total cholesterol
TG: Triglycerides
VIF: Variance inflation factor
VLDLc: Very-low-density lipoprotein cholesterol
χ^2^: Chi-squared
